# Cellular polyploidy in organ homeostasis and regeneration

**DOI:** 10.1093/procel/pwac064

**Published:** 2022-12-31

**Authors:** Juntao Fang, Alain de Bruin, Andreas Villunger, Raymond Schiffelers, Zhiyong Lei, Joost P G Sluijter

**Affiliations:** Department of Experimental Cardiology, University Medical Center Utrecht, Heidelberglaan 100, 3584 CX Utrecht, The Netherlands; Department of Pediatrics, University of Groningen, PO Box 30.001, 9700 RB Groningen, The Netherlands; Department of Pathobiology, Dutch Molecular Pathology Center, Utrecht University, Yalelaan1, 3584 CL Utrecht, The Netherlands; Institute for Developmental Immunology, Biocenter, Medical University of Innsbruck, Innrain 80, 6020 Innsbruck, Austria; Ludwig Boltzmann Institute for Rare and Undiagnosed Diseases, Lazarettgasse 14, AKH BT 25.3 c/o CeMM Research Building, Haupteingang Level 1, 1090 Wien, Österreich; CeMM Research Center for Molecular Medicine of the Austrian Academy of Sciences, Lazarettgasse 14, AKH BT 25.3, A-1090 Vienna, Austria; CDL Research, University Medical Center Utrecht, Heidelberglaan 100, 3584 CX Utrecht, The Netherlands; Department of Experimental Cardiology, University Medical Center Utrecht, Heidelberglaan 100, 3584 CX Utrecht, The Netherlands; CDL Research, University Medical Center Utrecht, Heidelberglaan 100, 3584 CX Utrecht, The Netherlands; Department of Experimental Cardiology, University Medical Center Utrecht, Heidelberglaan 100, 3584 CX Utrecht, The Netherlands

**Keywords:** cellular polyploidy, tissue regeneration, cardiac regeneration, liver regeneration

## Abstract

Polyploid cells, which contain more than one set of chromosome pairs, are very common in nature. Polyploidy can provide cells with several potential benefits over their diploid counterparts, including an increase in cell size, contributing to organ growth and tissue homeostasis, and improving cellular robustness via increased tolerance to genomic stress and apoptotic signals. Here, we focus on why polyploidy in the cell occurs and which stress responses and molecular signals trigger cells to become polyploid. Moreover, we discuss its crucial roles in cell growth and tissue regeneration in the heart, liver, and other tissues.

## Introduction

### Establishing cellular polyploidy

Polyploidy is a widespread cellular feature in nature and plays an essential role in organism growth. Eukaryotic organisms usually have two complete sets of homologous chromosomes, a eukaryotic cell typically duplicates its chromosomes to produce two equal diploid sets of the genome during S phase, which will evenly divide and generate two identical diploid daughter cells. In contrast, cells that harbor more than two complete sets of homologous chromosomes are considered to be polyploid. This can be observed either in cells with a single polyploid nucleus or in multinucleated cells with diploid or even polyploid nuclei. In plants, polyploidy is likely to affect their fitness, which helps them adapt to the changing environment and provides new genetic material to induce genetic variations, resulting in higher diversity or novel gene functions (Leitch and Leitch, 2008). Polyploidy is also common in insects and vertebrates (Bogart, 1979). In certain types of mammalian cells, such as mammary gland cells (Rios et al., 2016), subperineurial glia (SPG) cells (Unhavaithaya and Orr-Weaver, 2012), cardiomyocytes and hepatocytes (Anatskaya and Vinogradov, 2007), skin keratinocytes (Zanet et al., 2010), and placenta trophoblast giant cells (Sher et al., 2013), the transition from diploid to polyploid appears to be essential for cellular function during their postnatal development (Senyo et al., 2013; Wilkinson et al., 2019a). In specific tissues, the generation of polyploid cells is associated with different cellular stress, like mechanical or metabolic stress, which can be observed in the myocardium and vascular smooth muscle cells when exposed to pressure overload (Vliegen et al., 1995; Hixon et al., 2000). Interestingly, in humans, extra chromosomes, such as triploid and tetraploid fetuses, are usually aborted or die early after birth due to malformations (Jacobs et al., 1982; Guc-Scekic et al., 2002). Therefore, polyploidy only exists in some specific tissues rather than in the whole organism.

Many scientists tried to understand why cells become polyploid and proposed some hypotheses to explain this phenomenon. First, polyploid cells generally are larger than diploid cells, thereby contributing to organ growth and tissue homeostasis (Unhavaithaya and Orr-Weaver, 2012). Second, more genome copies can prevent the unfavorable consequence of mutations and DNA damage, especially in some key genes, such as tumor suppressor genes (Zhang et al., 2018a), thereby improving cellular responsiveness and cell survival in response to stress (Anatskaya and Vinogradov, 2007; Mehrotra et al., 2008). Third, cell division might be a potential threat under certain circumstances; for instance, skin keratinocytes and placenta trophoblast giant cells are essential in protecting tissue integrity, and cell division may temporarily impair this function (Zanet et al., 2010; Sher et al., 2013). Fourth, cells may preserve extra energy via cytokinesis failure (Anatskaya and Vinogradov, 2007). As every condition has pros and cons, there are also some potential drawbacks for being polyploid. For example, if polyploid cells try to complete mitotic division, gene copies and multiple centrosomes may lead to asymmetric distribution and genetic variation in daughter cells and trigger aneuploidy (Duncan et al., 2012a). There is strong evidence for a high frequency of aneuploidy in cancer, such as acute myeloid leukemia (Barnard et al., 2002) and Ewing’s sarcoma (Maurici et al., 1998). Unexpected tetraploidy is also observed in human cancers (Ganem et al., 2007). Tetraploidy is viewed as a metastable intermediate between healthy diploidy and neoplastic aneuploidy, and it has been observed in certain types of diseases like breast cancer (Jonsdottir et al., 2012) and Barrett’s esophagus (Reid et al., 1987). In short, polyploid can either contribute to important tissue function or, on the other hand, is associated with the development of cancer.

This review summarizes the formation of polyploidy and its essential roles in different organs and reveals a potential crosslink between polyploidy and tissue regeneration.

### Mechanisms for the genesis of polyploid cells

**Figure 1.**
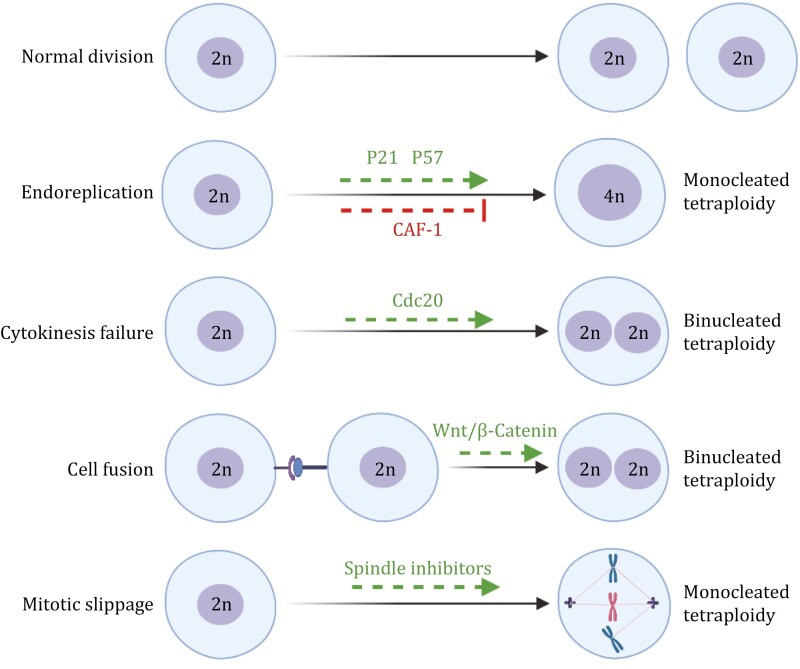
**Molecular mechanisms trigger polyploidy formation.** Different mechanisms are responsible for the genesis of different tetraploid cells. Cell fusion through receptor–ligand interactions or defective cytokinesis can form binucleated tetraploid cells. Endoreplication (cells skip mitosis) or mitotic slippage (cells exit mitosis without undergoing anaphase) generates mononucleated tetraploid cells. Note: Indicated mechanisms are only representative mediators and not a complete list.

Different mechanisms can convert diploid cells turn into polyploid cells. Different diploid cells can obtain a polyploidy state through various mechanisms, some even have more than one way to accomplish this state. Here we list four potential routes of polyploid cell formation: endoreplication, cytokinesis failure, cell fusion, and mitotic slippage, and all these mechanisms will be discussed in the following section (Fig. 1).

#### Endoreplication

Endoreplication, also known as endoreduplication or endocycles, occurs when cells successively copy their genomes without chromosome segregation, thereby leading to the genesis of mononucleated polyploid cells. The key event that triggers endoreplication is inhibition of entry into mitosis, usually mediated by mitotic cyclins under the control of the anaphase-promoting complex/cyclosome (APC/C) (Zielke et al., 2008). Endoreplication can also be induced by cyclin kinase inhibitors *P57* and *P21* via CDK1 activity suppression (Ullah et al., 2008; Ogawa et al., 2016). Large subunit of chromatin assembly factor-1 (CAF-1) is required for growth and larval viability, the depletion of the CAF-1 large subunit causes an accumulation of DNA damage, and an increased endoreplication, implying that endoreplication may be an adaptation to genomic damage (Klapholz et al., 2009), consistent with previous studies (Lazzerini Denchi et al., 2006; Mehrotra et al., 2008; Davoli et al., 2010). In Arabidopsis plants, the appearance of DNA damage led to G2–M arrest and ultimately triggered endoreplication, which is more tolerant to water deficit (Cookson et al., 2006). This means endoreplication can provide an advantage in adaptation to stress and improve growth in plants.

Tissue repair normally needs either cell growth or proliferation; when cell proliferation is limited, endoreplication ensues tissue repair can still continue. Forcing injured adult Drosophila abdominal epithelium to finish mitosis is unfavorable to wound repair because Drosophila epithelium can accumulate DNA damage, while mitotic errors occur when cells are compelled to proliferate (Grendler et al., 2019). In contrast, injury to Drosophila epithelium promotes cells to undergo endoreplication and cell fusion for wound healing (Losick et al., 2013; Grendler et al., 2019), indicating polyploidy can replace damaged cells and sustain normal cellular functions. This phenomenon also happens in the mouse model of Fuchs endothelial corneal dystrophy, where massive cell death drives pathology, and the remaining cells try to increase their ploidy status to compensate for cell loss and maintain size and synthetic capacity (Losick et al., 2016). Endoreplication is also critical to normal cell growth in some specific tissues. For example, reduced ploidy of ovarian nurse cells can lead to female sterility, as endoreplication is indispensable for normal oocyte development in Drosophila (Lilly and Spradling, 1996; Maines et al., 2004).

#### Cytokinesis failure

**Figure 2.**
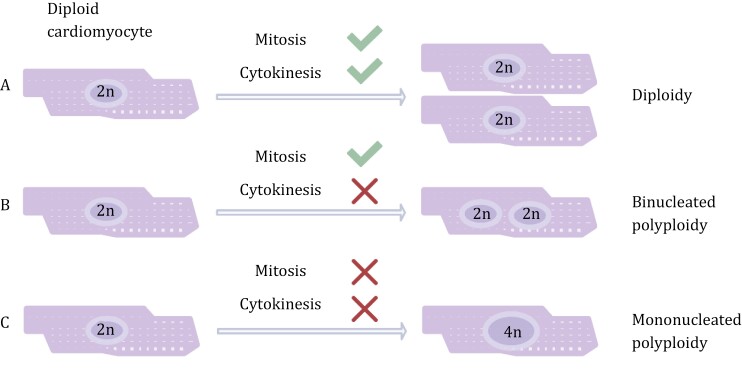
**The formation of polyploid cardiomyocyte during postnatal development.** Normally, cardiomyocytes can complete both mitosis and cytokinesis, leading to the formation of two diploid daughter cells (A). Once the cardiomyocyte completes only mitosis with defective cytokinesis, it will give rise to one binucleated cardiomyocyte (B). If the cardiomyocyte failed in both mitosis and cytokinesis, it would generate one polyploid cardiomyocyte nuclei (C)..

Cytokinesis is the last step in mitosis, promoting physical separation and driving the emergence of two daughter cells from one parental cell. Successful cytokinesis is required to distribute replicated genomes and other cellular components into subsequent daughter cells, while cytokinesis failure can give rise to binucleated polyploid cells and supernumerary centrosomes (Ganem et al., 2007; Rengstl et al., 2013). Although the reason for structural chromosome aberrations in polyploid cells remains unknown, improper chromosome segregation caused by extra centrosomes might be responsible for the aberrations (aneuploidy) often found in polyploid cells (Ganem et al., 2009). Cytokinesis failure occurs during the development of specific human organs, such as the heart or the liver (Fig. 2). Cytokinesis failure-induced polyploidy is also called endomitosis. Increased mechanical tension causes cytokinesis failure and generates polyploid cells in the epicardium, which are efficient enough to support epicardial regeneration (Cao et al., 2017).

#### Cell fusion

Cell–cell fusion is a special mechanism that creates polyploid cells without involving cell cycle progression. It is a crucial cellular process in which several mononuclear cells integrate to produce a multinucleated cell (Kemp et al., 2011, 2012). Cell fusion is also vital during tissue development and regeneration, as cytotrophoblasts fuse to generate placental syncytiotrophoblasts (Huppertz et al., 1998). *In vivo*, mouse retinal neurons can reach a precursor stage after spontaneously fusing with transplanted hematopoietic stem and progenitor cells via the activation of Wnt/β-catenin signaling, the newly formed cells subsequently proliferate and finally become differentiated neurons to rescue the damaged retinal tissue (Sanges et al., 2013). In our previous study, we observed that inhibition of bone marrow cells recruitment into the liver severely dampen its regeneration capacity following injury, where the mobilized hematopoietic stem and progenitor cells from resected liver were able to fuse with hepatocytes, and these hybrid cells proliferate earlier than hepatocytes (Pedone et al., 2017).

#### Mitotic slippage

During perturbed mitosis, when cells are subjected to spindle inhibitors, they are blocked at pro-metaphase by the spindle assembly checkpoint (SAC), which helps avoid chromosome mis-segregation and ensures each chromosome is properly attached to the mitotic spindle (Brito and Rieder, 2006; Mansilla et al., 2006). This blockage is caused by APC/C inhibition via the mitotic checkpoint complex, which is pivotal to regulate mitotic progression (Brito and Rieder, 2006). Normal cells have a robust SAC in which unattached chromosomes initiate signals to interfere with cellular APC/C activity and thereby hinder progression to anaphase (Brito and Rieder, 2006). When the SAC is weak, cells slip out of mitosis without undergoing anaphase or cytokinesis, known as mitotic slippage, which is prevalent in cells with prolonged mitotic arrest after exposure to spindle toxins treatment or in APC defective cells, and appear rarely spontaneous under physiological condition (Brito and Rieder, 2006; Dikovskaya et al., 2007). Microtubule-targeting agents that stimulate the SAC and disturb microtubule dynamics are able to cause cell death (Lamar et al., 2016; Kang et al., 2018). However, suppression of the SAC and subsequent slippage can lead to chromosome instability and is lethal to normal and tumor cells, demonstrating that its function is critical to cell survival (Kops et al., 2004; Michel et al., 2004).

## Polyploidy in the heart

### Cardiomyocyte polyploidy during development

The extent of cardiomyocyte polyploidy varies greatly in vertebrate species (Ostergaard et al., 2013; Bensley et al., 2016; Hirose et al., 2019). In endotherm mammalian and bird species, polyploid cardiomyocytes are the primary population (more than 90%), while most cardiomyocytes in zebrafish and newts are diploid (nearly 99%) (Bettencourt-Dias et al., 2003; Hirose et al., 2019). Of note, some fish species preserve substantial cardiomyocyte polyploidy (up to 85%) (Martynova et al., 2002). Noticeably, the nuclear number in polyploid cardiomyocytes varies among endotherm species (Adler et al., 1996; Anatskaya and Vinogradov 2004; Bensley et al., 2016). The majority of human adult cardiomyocytes only have one polyploid nucleus (about 60%) (Olivetti et al., 1996; Mollova et al., 2013). However, giraffe and bird cardiomyocytes are predominantly multinucleated, with 3–8 nuclei per cardiomyocyte, respectively (range from 50% to 99%; Anatskaya et al., 2001; Ostergaard et al., 2013; Hirose et al., 2019). Other mammalian species, such as mice, rabbits, sheep, and rats, displayed predominantly binucleated heart cells (range from 66% to 82%; Adler et al., 1996; Raulf et al., 2015; Bensley et al., 2016). In murine heart development, most cardiomyocytes (80%–95%) turn into polyploidy around 1 week after birth (Soonpaa et al., 1996). Interestingly, the presence of polyploid cardiomyocytes coincides with the change in thyroid hormone levels (Hirose et al., 2019). In sheep and mice, thyroid hormone plasma concentration increases several folds after birth, along with decreased cardiomyocyte proliferative capacity and increased cardiomyocyte polyploidy (Chattergoon et al., 2012a; Hirose et al., 2019). Additional thyroid hormone administration can significantly augment cardiomyocyte polyploidy; in contrast, inhibition of thyroid hormone receptors in cardiomyocytes of neonatal mice results in increased proliferative capacity and diploid cardiomyocyte number (Chattergoon et al., 2012a, 2012b; Hirose et al., 2019). This finding may explain why cardiomyocytes in some species become polyploid after birth.

During the perinatal period, cardiomyocytes are exposed to extensive oxidative stress, accompanied by increased DNA damage and cell cycle exit (Puente et al., 2014). DNA damage induced by oxidative stress causes mis-segregation of chromosomes during the M phase, which might be the reason for cardiomyocyte polyploidy (Puente et al., 2014; Aix et al., 2016). Besides, polyploidy in cardiomyocytes could be an adaptation to stress induced by increased hemodynamic demand in fetal or neonatal hearts (Barbera et al., 2000; Jonker et al., 2010). In the early stage of human heart development, most cardiomyocytes are diploid, where heart mass growth mainly depends on the proliferation of cardiomyocytes. However, shortly after birth, most cardiomyocytes lose the ability to complete cytokinesis and become binucleated, and the subsequent increase in heart size is independent of cardiomyocyte proliferation (Li et al., 1996; Soonpaa et al., 1996).

Interestingly, cardiomyocyte polyploidy appears even before birth in sheep (Jonker et al., 2007a). Thus, being born is unlikely to be the only reason for increased polyploid cardiomyocytes in mammals. Cell cycle regulators seem to be associated with cardiomyocyte polyploidy. Almost all cyclins are highly expressed during the embryonic stage, and subsequently decreases after birth (Kang et al., 1997), in particular, cyclin A and B expression levels are reportedly undetectable in adult hearts (Kang et al., 1997). The CDKs were also highly expressed during embryonic development while dropping to very low levels in the young hearts, consistent with the proliferating cell nuclear antigen (PCNA) pattern during this period (Kang et al., 1997), indicating that the decreased level of mitotic cyclins and CDKs probably trigger the withdrawal from cell cycle after birth, which ultimately triggers the occurrence of polyploid cardiomyocytes (Kang et al., 1997). Exogenous expression of mitotic cyclins seems a feasible way to overcome this hurdle. In the adult porcine heart, overexpression cyclin A2, a mitotic cyclin, in infarct myocardium caused increased cytokinesis and cardiomyocyte numbers with mature sarcomeric structure (Shapiro et al., 2014). Similarly, overexpression of cyclin B1-CDK1 complexes restarts cell division in adult rat cardiomyocytes (Bicknell et al., 2004). Conversely, CDK inhibition leads to the cell cycle exit of cardiomyocytes (Di Stefano et al., 2011; Tane et al., 2014). In addition, enhanced *E2f1* and *E2f2* can accelerate cell cycle and cardiomyocyte division via increased expression of cyclins B1 and B2. *Meis1* was also identified as a critical regulator of the cardiomyocyte cell cycle. *Meis1* silencing in mouse cardiomyocytes promotes extension of the postnatal proliferative window of cardiomyocytes and reactivates cardiomyocyte mitosis in the adult heart without detrimental effect on cardiac function. Instead, overexpression of *Meis1* in cardiomyocytes dampens neonatal cardiomyocyte proliferation capacity (Mahmoud et al., 2013). Below, we summarize some crucial genes and conditions that were known to influence polyploidy in the heart (Table 1).

**Table 1.** Summary of different genes and stimuli that alter polyploidy in heart.

Genes	Gene function	Manipulation	Polyploidy	Effect	Ref
*Myc*	Transcription factor, proto-oncogene	Overexpression	Increased	Maintain cardiac hypertrophy; Heart function unchanged	Xiao et al. (2001)
Cyclin G_1_	Cell cycle, G_2_/M arrest	Deletion/ Overexpression	Decreased/Increased	Decreased proliferation capacity	Liu et al. (2010)
Cyclin D_1_	Cell cycle, G_1_/S transition	Overexpression	Increased	Modest increased proliferation capacity	Soonpaa et al. (1997)
*Ect2*	Cell cycle, mitosis	Deletion	Increased	Decreased proliferation capacity	Gonzalez-Rosa et al. (2018)
*Tnni3k*	Cardiac physiology	Overexpression	Increased	Decreased proliferation capacity	Patterson et al. (2017)
*Lamin B2*	Cell mitosis, nuclear stability, chromatin structure	Deletion	Increased	Decreased proliferation capacity	Han et al. (2020)
*Fgl2*	Immune regulator	Deletion	Decreased	Decreased hypertrophy; Triggered early death	Fan et al. (2021)
Survivin	Cytokinesis regulation, cell apoptosis	Deletion	Increased	Decreased cell cycle progression	Levkau et al. (2008)
*Gsk-3*β	Energy metabolism, mitochondrial dysfunction	Deletion	Increased	Severe dilated cardiomyopathy	Zhou et al. (2016)
*Gas2l3*	Actin and microtubule regulation	Deletion	Increased	Decreased proliferation capacity and hypertrophy	Stopp et al. (2017)
Conditions					
Thyroid hormone		Inactivation	Decreased	Increased proliferation capacity	Hirose et al. (2019)
Hypoxia		Induction	Increased	Enhanced mitochondria function and less apoptosis	Jiang et al. (2020)
Tension		Induction	Increased	Increased regenerative capacity	Cao et al. (2017)
Pressure overload		Induction	Increased	Myocardial hypertrophy	Vliegen et al. (1991); Vliegen et al. (1995)

The centrosome is essential for separating duplicated DNA during cell division, acting as a critical hub for cell cycle regulation that determines if a cell should divide (Pascreau et al., 2011; Zebrowski et al., 2015). In adult newts and zebrafish, the structure of centrosomes within the heart muscle cells remains intact, while centrosomes in mammalian cardiomyocytes are disassembled shortly after birth, thereby potentially triggering P38-MAPK-mediated cell cycle arrest (Zebrowski et al., 2015). In contrast to this, P38-MAPK inhibition can promote the proliferation capacity of cardiomyocytes with split-centrioles, indicating that interference of *P38*-mediated stress-associated signal enhances cell cycle progression in cardiomyocytes that lack centriole cohesion (Zebrowski et al., 2015). Anillin is a cytokinesis-associated protein crucial for furrow construction and midbody formation during cytokinesis. P38-MAPK-mediated anillin localization defects create a barrier for midbody formation, leading to an incomplete cell cycle and binucleated cardiomyocytes, while P38-MAPK inhibition can repair the midbody formation failure (Engel et al., 2006). Similarly, Septins, a family of cytoskeletal GTPases, correlate with cytokinesis during heart development (Ahuja et al., 2006).

So far, the reason for cardiomyocyte polyploidy formation is still poorly defined. One hypothesis is that cardiomyocyte polyploidy is regulated as a response to cardiomyocytes’ physiological and functional demands. Cardiomyocytes may temporarily choose to undergo sarcomeres disassembly to meet the increased cardiac output. However, there are several potential drawbacks to this solution. First, it could be energy consuming and cause contractile dysfunction during cytokinesis. Consistent with this, upregulation of genes encoding contractile-related proteins was observed in polyploid heart tissues (Anatskaya and Vinogradov, 2007). Suppose the electric signal was conducted less homogenously and has to pass across more gap junctions; this could bring an increased risk of defective electrical coupling between newly developed cardiomyocyte and pre-existing cardiomyocyte (Gabisonia et al., 2019). Thus, cardiomyocyte polyploidy might be helpful as it contributes to heart growth and sustains myocardial contractile function. Furthermore, cardiomyocyte polyploidy contributes to coping with cellular stress, prolonging cell longevity by increased resistance to various stress, offering a faster transcriptional response to the changing physiological environments (Anatskaya and Vinogradov, 2007), and meeting increased protein synthesis requirements (Clubb and Bishop, 1984; Oparil et al., 1984; Soonpaa et al., 1996). In this case, cardiomyocyte polyploidy probably provides an alternative solution for increased heart mass from an energy-saving perspective. In brief, cardiac muscle structure stability, contractility capacity, and electrical coupling provide us with some viewpoints to comprehend why cardiomyocytes become polyploid during heart growth.

### Cardiomyocyte polyploidy in heart pathology

Increased cardiomyocyte ploidy can be observed both in physiological and pathological conditions (Adler and Friedburg, 1986; Bergmann et al., 2015; Gilsbach et al., 2018). Cardiomyocyte ploidy in rodents and humans increases with age (Soonpaa et al., 1996; Li et al., 1996; Bergmann et al., 2009; Bergmann et al., 2015). Interestingly, cardiomyocyte ploidy level was elevated (up to 25%) after myocardial infarction, especially in the infarcted border zone (Meckert et al., 2005; Hesse et al., 2012). Infarction likely activated the remaining cells’ DNA synthesis, providing an adaptive response to the damaged myocardium (Herget et al., 1997). This implies that cardiomyocytes have the potential capacity to reenter the cell cycle and undergo DNA replication under pathological conditions. In addition, cardiomyocyte ploidy levels are also increased (up to 15–20n) in cardiac hypertrophy and in adults with congenital diseases (Erokhina et al., 1992; Brodsky et al., 1994). Pressure overload-induced cardiac hypertrophy appears to be correlated with polyploidy and is more apparent in the left than the right ventricle (Vliegen et al., 1995). In order to explore the relationship between cardiomyocyte proliferation, size, and polyploidy under hypertension, Jonker et al. (2007b) treated fetal sheep with chronic intravascular plasma infusions for 4 or 8 days to induce hypertension. The incidence of cell cycle activity is higher in the hypertension group than the control group. In the early phase, cell cycle activity increased as well as cardiomyocyte enlargement, whereas, in the later phase, binucleate cardiomyocyte number elevated (approximately 50%) with enhanced cell cycle activity, suggesting there are different physiological responses to hypertension in the fetal sheep heart (Jonker et al., 2007b).

A recent article shows that running exercise stimulates physiological cardiac hypertrophy and facilitates cell cycle activity, leading to increased diploid cardiomyocytes (nearly double) and newborn cardiomyocytes, even in injured mice that underwent myocardial infarction (Vujic et al., 2018). So far, no relevant research has investigated whether exercise-mediated physiological hypertrophy in humans can also increase the percentage of diploid cardiomyocytes.

## Polyploidy in liver

### Polyploidy in liver during development

**Figure 3.**
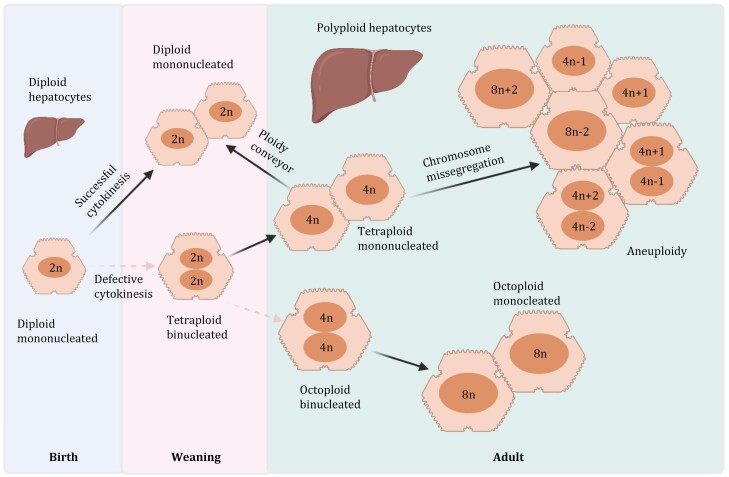
**Hepatocytes polyploidy during development.** Gradual hepatocyte polyploidy emerges in rodent during postnatal growth and is nearly diploid in newborns (1 × 2n). During weaning, diploid hepatocytes can either complete the cell cycle and produce two diploid hepatocytes or undergo incomplete cytokinesis and generate binucleated tetraploid hepatocytes (2 × 2n). Two mononucleated tetraploid cells are formed when binucleated cells enter the next cell cycle with normal cytokinesis. This continued process results in the formation of mono- or binucleated tetraploid and octoploid (8n) hepatocytes and so on. In the adult, hepatocytes regulate its ploidy by responding to different signals. They can either increase (such as cytokinesis failure) or decrease ploidy state (multipolar spindle formation, a process called ploid reversal). During this ploidy reversal process, chromosome segregation errors could happen, which triggers the formation of aneuploid cells.

The level of hepatocyte polyploidy varies among mammals, and polyploidy is seen as a critical feature of liver growth and physiological function (Anatskaya et al., 1994). Up to 90% of adult hepatocytes in murine and around 50% in adult humans are polyploid (Saeter et al., 1988; Duncan et al., 2010; Duncan et al., 2012b). Increased cell size appears to be an obvious result of an increase in ploidy, as it was usually accompanied by an increase in cell volume (Epstein, 1967). The volume of hepatocytes almost doubled with doubling DNA content. Interestingly, there is no dramatic difference between the volume of mononuclear and binuclear polyploid hepatocytes containing the same amount of DNA (2 × 2n and 4n cells) (Martin et al., 2002). One attractive question is how diploid hepatocytes become polyploid during physiological or pathological development. In the liver, polyploid cells appeared during suckling-to-weaning transition in which nutrition change is involved. This change will have an impact on liver metabolism, which might be involved in the formation of polyploid cells (Margall-Ducos et al., 2007; Celton-Morizur et al., 2009). During this period, diploid hepatocytes (2n) undergo cell cycle, producing two diploid hepatocytes or giving rise to one binucleate tetraploid hepatocyte because of failed cytokinesis. This process is a pivotal step for establishing polyploid hepatocytes during postnatal growth. When the binucleate tetraploid hepatocyte initiates the next cell cycle, it can initiate DNA replication again, leading either to successful cytokinesis with the generation of two mononucleate tetraploid hepatocytes (4n) or to defective cytokinesis with one binucleate octoploid hepatocyte generation (2 × 4n) (Guidotti et al., 1909). Polyploid hepatocytes contain extra centrosomes that can form multipolar spindles during mitotic division, thereby producing progeny with reduced ploidy. In this process, frequent chromosome mis-segregation occurs and causes structural rearrangements of chromosomes and the formation of aneuploid hepatocytes (Fig. 3). Guidotti *et al.* (1909) demonstrated that cytokinesis failure might derive from deficiencies of actin cytoskeleton reorganization. Consequently, astral microtubules cannot contact the cortex and convey molecular signals properly, interfering with the activation of the *RhoA* pathway, which causes cytokinesis defect and binucleated tetraploid hepatocyte formation (Margall-Ducos *et al.*, 2007).

Insulin has been thought as a critical regulator in the appearance of binucleated polyploid hepatocytes. How does insulin regulate polyploidy development? Insulin signaling inhibition drastically hindered the formation of binuclear polyploid hepatocytes, whereas injection with insulin promoted the generation of polyploid cells (Nakatani et al., 1997; Gorla et al., 2001). Evidence shows failed cytokinetic events in hepatocytes after weaning might be modulated by insulin via PI3K-AKT pathway (Celton-Morizur et al., 2009; Celton-Morizur et al., 2010). AKT activity inhibition in hepatocytes boosts the actin cytoskeleton reorganization during mitosis and promotes successful localization of *RhoA* to the dividing area, which is required for cytokinesis (Celton-Morizur et al., 2009; Yoshida et al., 2009; Celton-Morizur et al., 2010). Although cytokinesis failure is a crucial mechanism for hepatocyte polyploidy, cell fusion was also observed in this process and can be one possible explanation for the formation of polyploid hepatocytes during postnatal liver growth (Faggioli et al., 2008; Faggioli et al., 2011). Further research suggests that hepatocyte–hepatocyte fusion in adult mice is negligible (Willenbring et al., 2004).

Oxidative damage may be another mechanism for hepatocytes polyploidization, a mechanism also discussed in heart ploidy control. Oxidative stress induced by pathological changes in nonalcoholic fatty liver activates G_2_/M DNA damage checkpoint, eventually leading to highly polyploid hepatocytes. However, antioxidant treatment blocks the initiation of the G_2_/M DNA damage checkpoint, restoring normal cell division and returning to a normal ploidy profile (Gentric et al., 2015). Oxidative DNA injury induced by radiation exposure and antioxidant depletion causes an increase in polyploid hepatocytes number and impair cellular proliferation capacity (Gorla et al., 2001; Malhi et al., 2002), in line with Nakatani’s study that endogenous antioxidant enzymes overexpression in transgenic mice significantly reduced hepatocyte polyploidy (Nakatani et al., 1997).

In addition to the insulin pathway and oxidative stress, other factors are also involved in the modulation of hepatic polyploidy. Table 2 summarizes key genes and stimuli that either promote or hinder polyploidy formation in the liver. In adults, hepatocyte polyploidy is also associated with other cellular stress, including metabolic overloads such as iron or copper (Madra et al., 1995; Muramatsu et al., 2000), telomere dysfunction (Lazzerini Denchi et al., 2006), and chronic hepatitis B/C infection (Toyoda et al., 2005; Toyoda et al., 2006). All these findings indicated a strong correlation between the genesis of polyploid hepatocytes and cellular stress, providing new insight for comprehending liver development and regeneration. Besides, *E2f7*/*8* transcription factors are essential for hepatocyte polyploid. One astonishing finding is that loss of *E2f7*/*8* has no impact on liver differentiation, metabolism, and regeneration (Pandit et al., 2012), raising the possibility that polyploid is dispensable for normal hepatocyte function. *E2f7*/*8* also controls PIDDosome function, which is critical to hepatocyte polyploid regulation (Sladky et al., 2020a; Sladky et al., 2021). The PIDDosome is a protein complex containing the death domain-containing protein PIDD1 and the adaptor protein RAIDD, both required to activate CASP2 in response to centrosome amplification (Tinel and Tschopp, 2004; Sladky and Villunger, 2020). Extra centrosomes activate the PIDDosome–p53–p21 axis in hepatocytes after the failure of cytokinesis (Sladky et al., 2020a). After PIDDosome formation is initiated, CASP2 is activated which inactivates the E3 ligase MDM2 to stabilize *P53* and its target gene *P21*. Consequently, this will lead to cell cycle arrest to limit hepatocyte polyploidy (Oliver et al., 2011; Fava et al., 2017; Sladky et al., 2020a). Consistently, the loss of CASP2, PIDD1, or RAIDD resulted in increased hepatocyte polyploidy (Sladky et al., 2020a), and protection from hepatocellular carcinoma, which indicates the PIDDosome might be a potential therapeutic target to control hepatocyte polyploidy for hepatocellular carcinoma prevention (Sladky et al., 2020b). Recent findings show that extra centrosomes are indeed the cue activating the PIDDosome, as hepatocytes lacking the master regulator of centriole biogenesis, *Plk4*, show also increased ploidy, as do hepatocytes that lack the connecting element between centrosomes and PIDD1, that is, *Ankrd26* (Sladky et al., 2022).

**Table 2.** Summary of key genes and virus factors known to affect polyploidy in liver.

Genes	Gene function	Manipulation	Polyploid	Effect	Ref
*c-Myc*	Transcription factor, proto-oncogene	Overexpression	Increased	Increased proliferation capacity	Conner et al. (2003)
*E2f1*/*2*/*3*	Cell cycle transcription factor	Deletion	Increased	Increased proliferation capacity	Chen et al. (2012)
*E2f7*/*8*	Cell cycle transcription factor	Deletion	Decreased	Unaffect differentiation/regeneration, spontaneous liver cancer	Pandit et al. (2012; Chen et al. (2012); Kent et al. (2016)
*Rb1*	Cell cycle, tumor suppressor	Deletion	Increased	Biochemically normal and without hyperplasia	Mayhew et al. (2005)
*Trp53*	Cell cycle transcription factor, tumor suppressor	Deletion	Increased	Prolonged proliferation capacity	(Kurinna et al. (2013)
CDK1	Cell cycle, mitosis	Deletion	Increased	Unimpaired regeneration capacity	Diril et al. (2012)
Survivin	Cell cycle, mitosis	Deletion	Increased	Impaired regeneration capacity	Hagemann et al. (2013); Li et al. (2013)
*Ercc1*	Cell cycle, DNA repair	Deletion	Increased	Increased oxidative damage and aging	Chipchase et al. (2003)
*Cdkn1a*	Cell cycle, DNA damage	Overexpression	Increased	Impaired regeneration capacity	Wu et al. (1996)
*Mapk14* (*P38alpha*)	Inflammation, immunity, cell death	Deletion	Increased	Decrease liver growth and life-span	Tormos et al. (2013, 2017)
*Skp2*	Cell cycle, mitosis	Deletion	Increased	Impaired regeneration capacity	Minamishima et al. (2002)
*miR-122*	Fatty acid metabolism, hepatitis	Deletion	Decreased	Promoted cytokinesis progression	Hsu et al. (2016)
*Ccne2*	Cell cycle, cancer	Deletion	Increased	Decreased proliferation capacity	Nevzorova et al. (2009)
*Ssu72*	Chromatin Regulation/Acetylation	Deletion	Increased	Increased liver injury	Kim et al. (2016)
TGF-β	Cell growth, proliferation, differentiation	Deletion	Decreased	Unaffect proliferation and hepatic index	De Santis Puzzonia et al. (2016)
*Atp7b*	Copper homeostasis	Deletion	Increased	Accumulative copper cytotoxicity	Muramatsu et al. (2000)
*Trf2*	Cell cycle, telomere protection	Deletion	Increased	Unaffect liver function and regeneration capacity	Lazzerini Denchi et al. (2006)
*Lrpprc*	Cytoskeletal and transcription regulation	Deletion	Increased	Impaired autophagy maturation	Li et al. (2020)
*Anln*	Cytoskeletal organization	Deletion	Increased	Unaffect liver regeneration	Zhang et al. (2018b)
*Atg7*	Cell recycling	Deletion	Increased	Trigger hepatomegaly and liver tumors	Kern et al. (2016)
*Lkb1*	Cell polarity, tumor suppressor	Deletion	Increased	Trigger carcinogenesis	Werle et al. (2014)
*Notch3*	Cellular differentiation, proliferation	Overexpression	Increased	Decreased proliferation capacity	Ortica et al. (2014)
*Csn*	Cellular developmental process	Deletion	Increased	Trigger cell death	Panattoni et al. (2014)
PIDDosome	Cellular differentiation, proliferation	Deletion	Increased	Increased proliferation capacity	Sladky et al. (2020a)
*Plk4*	Cellular differentiation, proliferation	Deletion	Increased	Increased proliferation capacity, increased damage upon repeat challenge	Fava et al. (2017)
*Ankrd26*	Cellular differentiation, proliferation	Deletion	Increased	Increased proliferation capacity, increased damage upon repeat challenge	Burigotto et al. (2021)
Conditions					
Partial hepatectomy		Two-thirds resection	Increased	Decreased proliferation capacity; trigger cell aging	Sigal et al. (1999)
Nonalcoholic fatty liver		Induction	Increased	Associated with hepatocellular carcinoma	Gentric et al. (2015)
Iron overload		Iron diet	Increased	Trigger hepatomegaly	Troadec et al. (2006)
Viral hepatitis		Hepatitis B or C virus	Increased	Increased fibrosis	Toyoda et al. (2005, 2006)
Thyroid hormone		Induction	Increased	Increased proliferation capacity	Gujabidze and Rukhadze (2006)

### Polyploidy produces genetic diversity in liver

Diploid hepatocytes can become polyploid by increasing their DNA content; however, polyploid hepatocytes can also develop multipolar mitotic spindles and form lower ploid hepatocytes. In some cases, cell division can produce offspring with lower ploidy states referred to as “ploidy reduction” (Guidotti et al., 1909; Duncan et al., 2010), which describes that hepatocytes can increase and then decrease their ploidy subsequently (Fig. 3). Although only a tiny proportion of polyploid hepatocytes might undergo ploidy reversal *in vitro* (Duncan et al., 2010), this seems to happen more extensively *in vivo*, where most polyploid hepatocytes experience ploidy reversal when forced to divide (Duncan *et al.*, 2010). A multipolar mitotic spindle formation is often associated with chromosome segregation errors. In this case, the division of polyploid hepatocytes caused random and unbiased whole chromosome gains/losses followed by aneuploidy emergence (Duncan et al., 2010; Faggioli et al., 2011; Duncan et al., 2012b) (Fig. 3). This unique feature is relevant to cells continuously challenged by stress, which may lead to genomic instability (Faggioli et al., 2011). Therefore, ploidy reversal presumably creates genetic diversity and contributes to hepatocyte adaptation in response to a xenobiotic or nutritional injury (Duncan et al., 2010, 2012b), thereby reducing polyploid hepatocyte proportion to match the low level of the youthful liver and augmenting the proliferative capacity (Wang et al., 2014).

Genomic instability has long been considered a cause of cancer. For this reason, it was a surprise that the liver contains a significant number of aneuploid cells (Duncan et al., 2010, 2012b). Aneuploidy appeared randomly in healthy mice as all chromosomes were affected equally (Duncan et al., 2012a). Aneuploidy is also found in many healthy human tissues lacking evidence of malignancy, including skin, brain, ear, and kidney (Turker et al., 2004; McConnell et al., 2013; Knouse et al., 2014). Although aneuploidy is strongly correlated with cancer occurrence, Fujiwara *et al.* (2005) report that spontaneous liver cancer happens due to aneuploidy is quite rare (Chandra and Frith, 1992), challenging the concept of a causal link between aneuploid and cancer. Polyploid hepatocytes even facilitate liver regeneration after injury while undergoing ploidy reduction and subsequent re-polyploidy (Matsumoto et al., 2020). These findings further suggest that hepatocyte’s ploidy reversal and aneuploid were features of the normal liver. In some cases, aneuploidy seems to be a mechanism of adaptation to liver injury associated with the metabolic disorder (Duncan et al., 2012a). The loss of polyploidy in the liver resulted in decreased aneuploidy and impaired their ability to adapt to tyrosinemia-induced liver failure (Wilkinson et al., 2019a), indicating that polyploid hepatocytes are needed for the formation of aneuploidy offspring and is an important modulator that facilitates adaptation to chronic liver disease.

Up to now, it remains unclear how aneuploidy affects liver fate. Polyploid hepatocytes are less likely to be influenced by the gain/loss of one or more chromosomes, while aneuploid hepatocytes with two incomplete sets of chromosomes might change cellular function. For instance, losing one single homolog may create substantial genetic diversity. Some of the genes are monoallelically expressed, which might imply that chromosomal monosomies may result in the functional deprivation of many genes (Gimelbrant et al., 2007). Future studies are needed to explore the molecular mechanisms that control the ploid conveyor and how hepatocyte aneuploidy influences human health in physiological or pathological conditions.

### Polyploidy enhances liver functions

What is the advantage of polyploidy over diploidy during liver growth and regeneration? Numerous hypotheses have been proposed to account for the functional significance of polyploidy in the liver. The first hypothesis was that hepatocyte polyploidy is relevant to terminal differentiation and aging (Sigal et al., 1995). However, hepatocytes can undergo ploidy reversal, indicating that hepatocyte polyploidy does not necessarily mean cellular senescence. For instance, tetraploid hepatocytes still sustain highly regenerative capacity after partial hepatectomy (Miyaoka et al., 2012), and FACS-isolated polyploid hepatocytes proliferated extensively *in vivo* transplantation and *in vitro* studies (Overturf et al., 1999; Weglarz et al., 2000; Duncan et al., 2009). Accordingly, aging and terminal differentiation fail to accurately describe the appearance of polyploid hepatocytes. The second consideration is that hepatocyte polyploidy might be a protective mechanism against oxidative stress (Lu et al., 2007). Antioxidants are linked to a lower degree of polyploidy in the liver (Sanz et al., 1995), while oxidative injury can directly induce hepatocyte polyploidy (Gorla et al., 2001). Additionally, a series of genes are related to hepatocyte polyploidy; most of these genes reflect metabolism changes and DNA damage responses to oxidative stress (Anatskaya and Vinogradov, 2007; Lu et al., 2007). This supports the view that hepatocyte polyploidy is a normal process that is helpful to oxidative stress resistance and maintains the detoxification capacity. However, a study in yeast suggested that increased ploidy does not provide an extra benefit for survival under the condition of DNA damage agents (Mable and Otto, 2001). Another assumption for hepatocyte polyploidy might result from a positive response to postnatal growth rate and metabolic load. For instance, hepatocyte polyploidy increased when the growth rate is stimulated by thyroid hormone additives (Torres et al., 1999). Thus, possibly when resources are limited, it can channel the energy that may flow into cell division toward other purposes (Anatskaya et al., 1994; Vinogradov et al., 2001). This might be particularly favorable to rapid-growing tissues as it could facilitate adaptation to different stresses. Correspondingly, high-energy consumption was found in parallel to the changes in hepatocyte polyploidy during the suckling–weaning period (Celton-Morizur et al., 2009).

Studies in the mammalian heart and liver show a link between polyploidy and coordination of functional gene expression, which could promote cell survival and tissue regeneration under stressful conditions (Sher et al., 2013). Polyploidy can alter the gene expression profile that may contribute to improving liver function as polyploidy might enhance translation, for example, providing twice as many genes might generate twice as many proteins. However, this is not always the case. Polyploidy can also promote nonuniform genome, transcriptome, and metabolome alterations, which probably get things complicated (Schoenfelder and Fox, 2015). On the other hand, polyploid cells are generally twice as big, so twice as many genes generate twice as many proteins in twice as much cellular volume. Therefore, this may not have any real impact compared to two diploid cells. Microarray analysis was conducted with the same amounts of RNA from diploid, tetraploid, and octoploid hepatocytes of mice and demonstrated that no major changes in gene expression patterns among different ploidy hepatocytes could be observed (Lu et al., 2007). Gene expression profiles vary between diploid and mononucleate polyploid hepatocytes but not between diploid and binucleate polyploid hepatocytes (Kreutz et al., 2017), indicating that nuclear ploidy in liver influences hepatocyte fate rather than the total ploidy within a cell. Intriguingly, low- and high-ploidy hepatocytes are equally sensitive to CCl4-induced cell death. Since hyperpolyploid hepatocytes are larger, their death results in a greater loss of functional parenchyma (Sladky et al., 2022).

## Polyploid in other organs

### Retinal pigmented epithelial cells

Nearly 3% retinal pigmented epithelial (RPE) cells in humans are binucleated, the majority are multinucleated, which is in line with rod and cone photoreceptor density (Starnes et al., 2016). In rodents, the proportion of multinucleated RPE cells is more than 80%, and the amount increases with age (Chen et al., 2016). Interestingly, the anatomical region seems to be the critical reason for the number of multinucleated RPE cells in mice rather than age. The multinucleated RPE is related to the rods’ ratio, which could be an adaption to nocturnal vision. Multinucleated RPE cells exhibited elevated ROS production and DNA damage following irradiation in rodents (Ke et al., 2022), in line with previous research that multinucleated human RPE1 cells presented more γH2Ax-marked DNA damage (Hart et al., 2021). So far, the potential mechanisms for increased DNA damage in multinucleated RPE remain unknown.

Evidence revealed that newly developed RPE cells are mainly derived from remaining peripheral RPE cells, similar to the observation in cardiomyocytes (Hanovice et al., 2019). The majority of diploid RPE cells are found in the periphery of the retinal eye cup, and this proportion gradually drops when it moves to the central area, where polyploid cells expand with age (Al-Hussaini et al., 2008; Chen et al., 2016). Interestingly, the renewal of RPE cells either in physiological conditions or after an injury is mostly confined to the peripheral territory in which diploid RPE cells reside instead of the central area where polyploid RPE cells exist, suggesting that there might be a correlation between DNA content and regenerative capacity (Al-Hussaini et al., 2008; Chen et al., 2016).

### Pancreatic β-cells

The pancreas exerts a crucial role in regulating carbohydrate metabolism. Notably, polyploid in the pancreas occurs after weaning, coinciding with the adaption to hypertrophic growth (Finegood et al., 1995; Matondo et al., 2018). Pancreatic β-cells belong to endocrine cells and are surprisingly similar to cardiomyocyte division, as they are tightly interconnected and electrically excitable. Cell division in these cells probably impairs the interconnection and normal organ function (Meissner, 1976). As mentioned above, *E2f7*/*8* are pivotal regulators of hepatocyte polyploidy. To determine if *E2f7*/*8* can regulate the formation of polyploid cells in the pancreas, we used tamoxifen-inducible mouse models to assess the effect of postnatal *E2f7*/*8* gene deletion. To our surprise, decreased polyploidy, caused by *E2f7*/*8* genes deletion, did not show any striking consequence on postnatal growth and survival as well as the production of pancreatic hormones (Matondo et al., 2018). Instead, *E2f1*/*2*-deficient mice are more likely to have increased polyploidy, diabetes, and exocrine pancreas dysfunction (Iglesias-Ara et al., 2015). Loss of *P53* inhibited cell apoptosis and improved pancreas dysfunction and diabetes in mice lacking *E2f1*/*2*, indicating that *P53* may be required for tissue homeostasis and tumorigenesis prevention (Iglesias-Ara et al., 2015).

No convincing evidence revealed the link between DNA content and proliferative capacity in pancreatic cells. Nevertheless, a higher percentage of polyploid β-cells were observed in hyperglycemic states, which was interpreted as reflecting premature aging of β-cells (White et al., 1985). In addition, endoreplication-based hypertrophy without cell division was insufficient to maintain β-cell mass and compensate for increased metabolic demands (Zhong et al., 2007), suggesting that increased polyploid in β-cells may partly impair their proliferative capacity.

### Keratinocytes

By investigating the underlying mechanism of the formation of keratinocyte polyploidy, researchers observed that oncogenic alterations such as *P53* suppression, or genotoxic drugs can promote DNA damage that ultimately results in terminal differentiation and polyploidy (Di Micco et al., 2006; Freije et al., 2014). Continuous activation of *c-Myc* leads to mitosis block, endoreplication, and polyploidy in keratinocytes by inducing Cyclin E (Gandarillas et al., 2000; Freije et al., 2012). Inactivation of CDK1 or kinases of the mitosic spindle checkpoint, Aurora B or Aurora A, cell cycle kinases that can trigger keratinocyte terminal differentiation and polyploidy (Freije et al., 2012; Wen et al., 2012; Perez de Castro et al., 2013). Human papillomavirus type 16 proteins E6 and E7 can induce centrosome amplification and mis-segregation of chromosomes during mitosis, and eventually result in polyploidy in primary human keratinocytes, which may attribute to the disruption of the spindle checkpoint (Patel et al., 2004). The most attractive issue is why keratinocytes would accept to become polyploid and whether polyploidy endows additional functional support for the epidermis. Possibly, polyploid cells present stronger resistance than diploid cells in response to mechanical stress, and the rigidity given by keratins may restrict normal cytokinesis leading to cell division failure, similar to sarcomeric actin in the heart. As we know, keratinocytes are more exposed to harmful radiation. Therefore, it would not be surprising that keratinocytes attempt to rescue genomic instability by adopting a polyploid strategy.

### Mammary gland cells

The mammary gland is a unique model to study how an organ alters its structure to match its physiological demands. At the beginning of gestation in mice, mitotic activity in the epithelial cells of the mammary gland is elevated, and the number of binucleated epithelial cells increases from the 18th day of gestation. From the 3rd to 18th day of lactation, the nuclear volume in mononucleated and binucleated epithelial cells increases, accompanied by higher output of mammary gland cells (Kriesten, 1984). Earlier findings (Banerjee et al., 1971; Banerjee and Wagner, 1972) indicated that DNA synthesis proceeds in the absence of commensurate cell division during late pregnancy and lactation, which is critical to the functional differentiation of the mammary epithelium. Milk production in nulliparous mouse mammary epithelium heavily relies on epithelial DNA synthesis capacity (Vonderhaar et al., 1978). Later studies suggested that mitotic events are irrelevant to the interdependence of DNA synthesis and milk production in nulliparous mouse mammary epithelium (Smith and Vonderhaar, 1981). Polyploid cells are also observed in lactating mammary glands in different species, including humans, cows, seals, and wallabies (Rios et al., 2016). Most of the secretory alveolar cells are binucleated, which occurs in the late stage of pregnancy because of cytokinesis failure. In mice, Aurora kinase A and *Plk1* can induce the formation of polyploid cells via defective cytokinesis during the lactogenic switch. The number of polyploid cells decreases with the deprivation of Aurora kinase A signal resulting in decreased milk production (Rios et al., 2016), suggesting that polyploid mammary epithelial cells facilitate milk production and augment the survival of offspring, consistent with Smith’s study (Smith and Medina, 1988) that DNA synthesis and lactation are linked.

### Subperineurial glia cells

Regulation of cell size is pivotal in organ development. The size of the nervous system increases dramatically during Drosophila larval development, while the number of SPG cells stays the same, most SPG cells become polyploid, and appear to be responsible for the growth of the nervous system (Unhavaithaya and Orr-Weaver, 2012). Inhibition of DNA replication could reduce SPG cell polyploidy and cell size, causing rupture of the septate junctions required for the blood–brain barrier formation, indicating that increased SPG cell size induced by polyploidy is needed to sustain a functional blood–brain barrier (Unhavaithaya and Orr-Weaver, 2012). miR-285 can decrease the expression of its downstream target cyclin E in SPG and triggers abnormal endoreplication, eventually leading to an aberrant DNA ploidy state and defective septate junctions (Li et al., 2017). Interestingly, SPG underwent two cell cycle changes during development, inducing endoreplication but restoring nuclear division capacity to increase nuclear content, which contributes to an increase in cell size (Von Stetina et al., 2018). Polyploid appears to be a tactic to coordinate tissue growth during organ development. The growing rate of DNA synthesis meets the increased metabolic demands as SPG gets larger during development (Unhavaithaya and Orr-Weaver, 2012). However, no clear relationship between cell size and DNA content was observed among different segmental nerves in SPG, indicating that this regulation might not be stringent as expected (Zulbahar et al., 2018).

## Regeneration

### Polyploid and heart regeneration

**Figure 4.**
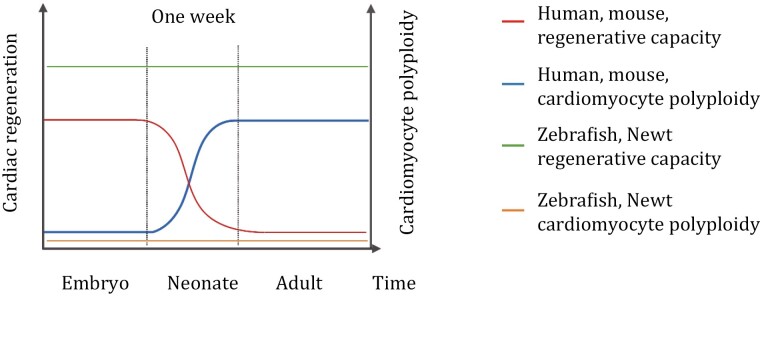
**Heart regenerative capacity in relation to cardiomyocyte polyploidy in different species with time.** Zebrafish and newt cardiomyocytes are exclusively diploid and maintain robust cardiac regenerative capacity throughout life. In contrast, cardiomyocyte polyploidy in humans and mouse increases with time, accompanied by the loss of heart regeneration capacity, suggesting that the occurrence of polyploidy might be at the cost of regeneration capacity decline in mammals.

The adult mammalian heart is a postmitotic organ and incapable of regeneration after injury (Li et al., 1996; Soonpaa et al., 1996). However, some evidence challenged this dogma and showed a low level of endogenous turnover of cardiomyocytes, estimated at around 1% per year in both adult mice (Senyo et al., 2013; Ali et al., 2014) and humans (Bergmann et al., 2009), even displaying a slight increase after myocardial infarction (Beltrami et al., 2001). The adult mammalian cardiomyocyte can hardly divide, but some vertebrates can. Adult zebrafish and newts can regenerate damaged hearts via cardiomyocyte division (Oberpriller and Oberpriller, 1974; Poss et al., 2002; Wills et al., 2008). Cardiomyocytes in the embryonic heart of mice are diploid and proliferative, whereas neonatal mice can also produce new cardiomyocytes within the first week after birth through the division of pre-existing cardiomyocytes. Subsequently, most cardiomyocytes undergo cytokinesis failure, leading to increased binucleated polyploid cardiomyocytes, accompanied by the loss of regenerative ability in the heart (Soonpaa et al., 1996; Porrello et al., 2011; Senyo et al., 2013). One major difference between these species is the ploidy of cardiomyocytes. Zebrafish and newt cardiomyocytes are exclusively diploid throughout life (Patterson et al., 2017), whereas adult mammalian cardiomyocytes are primarily polyploid, which occurs 1 week after birth (Soonpaa et al., 1996). Taken together, it seems that the transition of mouse cardiomyocytes from diploid to polyploid coincides with the loss of heart regeneration potential, similar to the observations in the human heart (Mollova et al., 2013; Ye et al., 2016) (Fig. 4). Of course, many other cellular changes emerge in the same neonatal window, including a rapid metabolic switch from glycolysis to fatty acid oxidation, an increase in oxygen consumption (Bartelds et al., 2000). So far, little is known about how these events contribute to cell cycle arrest and eventually result in cellular polyploidy.

Regenerative properties of the heart in mice largely rely on the number of diploid cardiomyocytes based on the analysis of the regenerative potential in different mouse strains (Patterson et al., 2017), in line with previous observation (Bersell et al., 2009). These findings led to the assumption that ploidy may play a crucial role in cardiomyocyte proliferative potential, further confirmed by several experimental approaches. A transgenic strategy by transient cytokinesis inhibition, modulated by dominant-negative *Ect2*, was used to induce a high degree of polyploid cardiomyocytes in zebrafish. Cardiomyocytes remained polyploid even after removing *Ect2* repression. Gonzalez-Rosa et al. (2018) demonstrated that conversion from diploid to polyploid in cardiomyocytes hindered regenerative capacity. Consequently, zebrafish are not able to regenerate injured hearts effectively or undergo ploidy reversal.

Similarly, in another study (Patterson et al., 2017), researchers observed that *Tnni3k* knockout mice presented a higher number of diploid cardiomyocytes as well as strengthened heart regeneration potential, while overexpression of *Tnni3k* in zebrafish promoted cardiomyocyte polyploid and compromised the capacity to regenerate, indicating that a critical proportion of diploid cardiomyocytes is needed to support heart regeneration. Inhibition of thyroid hormone receptors increased the number of diploid (nearly 2-folds) cardiomyocytes and improved myocardial regenerative ability, whereas animals treated with T3 hormone increased the percentage of binucleated cardiomyocytes (around 3-folds), followed by an interrupted cardiac regeneration (Hirose et al., 2019). All these results demonstrated compelling evidence that polyploidy creates a barrier to heart regeneration. However, ploidy is unlikely to be the only determining factor of heart regeneration because medaka fish could not achieve heart regeneration after myocardial injury with diploid cardiomyocytes (Lai et al., 2017).

Notably, these studies did not rule out the possibility that polyploid cardiomyocytes might also be able to support heart regeneration with appropriate stimulation. Several studies showed that binucleated cardiomyocytes can undergo complete cell division (Wang et al., 2017; Leone and Engel, 2019). Binucleated cardiomyocytes can be genetically driven to complete cytokinesis via β-catenin-induced expression of *Ect2* (Jiang et al., 2019). Engel et al. (2005) demonstrated that binucleated cardiomyocytes can divide into two mononucleated after being stimulated by P38-MAPK inhibition. In his study, a cell-tracing experiment was performed by utilizing cultured adult newt cardiomyocytes, which revealed that multinucleated cells have the capacity for cytokinesis and mitotic division. Some of the cells that undergo one complete cycle can also progress through several rounds of successful cell division (Bettencourt-Dias et al., 2003). This was consistent with D’Uva’s findings which were based on live-cell imaging of neonatal mouse cardiomyocytes and showed that 3.9% of binucleated cardiomyocytes can form two mononucleated daughter cells via *Erbb2* (D’Uva et al., 2015). However, this may challenge the Bersell *et al.*’s (2009) observation that only mononuclear cardiomyocytes could complete cytokinesis after Neuregulin 1 induction, rather than binucleated polyploid cardiomyocytes. All these evidences illustrate that binucleated cardiomyocytes still preserve proliferation potential. Nonetheless, it is still poorly understood if mononucleated polyploid cardiomyocytes can proliferate and how many binucleated cardiomyocytes can be induced to divide. Of note, these observations are based on an *in vitro* microscopy study, which is a reliable way to detect cell division through cytokinesis. However, cell culture lacks interaction with other cells and tissues, which may also affect cardiomyocytes’ senescence state, possibly making them behave differently when tested *in vivo*.

Mechanical stretching of epicardial cells can also induce endoreplication through tension and promotes regeneration of epicardial tissue (Cao et al., 2017). In these endoreplicating polyploid cells, the formation of cleavage furrow works normally, while tension causes defective cell abscission during cytokinesis, impairing midbody formation and abscission of the cleavage furrow, consistent with the previous observation that tension hinders cytokinetic abscission by perturbing the assembly of the ESCRT-III complex which is essential for successful abscission (Herget et al., 1997). Moreover, those polyploid cells appear to repair injured hearts more efficiently than diploid cells. In zebrafish, cardiomyocytes do not display polyploidy but upon surgical resection they disassemble their sarcomeric structure and detach from one another, attempting to facilitate cell cycle reentry (Duncan et al., 2012b). Genome-wide transcriptome profile analysis further confirmed this with the finding that the downregulation of sarcomeric-associated genes occurs after injury (Saeter et al., 1988). Newt hearts also undergo a massive downregulation of sarcomeric genes following amputation. As heart regeneration proceeds, the sarcomeric gene expression rises to normal levels 14 days after damage (Duncan et al., 2010). Although this disassembly process might cause mechanical uncoupling among cardiomyocytes and possibly impair the myocardial contractile power, the regenerative capacity in zebrafish and newts is still not compromised, which might attribute to their low blood pressure demands.

In conclusion, the biological function of cardiomyocyte polyploidy for heart development and regeneration is still unclear. Are the underlying mechanisms of polyploid cardiomyocytes the same during injury and homeostasis? What kind of characteristics are inherited by the daughter cells? Are there other different behaviors between polyploid and diploid cardiomyocytes? More investigations are therefore required to elucidate these issues in the future. One more important thing that needs to be noticed is, the traditional markers of cardiomyocyte proliferation applied in these studies, such as Ki67, pHH3. and Aurora B Kinase are inadequate to claim authentic cardiomyocyte proliferation. Ki67 is a marker indicating cell cycle progression (Gerdes et al., 1983; Duchrow et al., 1996) and used to be a compelling marker for assessing cellular proliferation. However, Ki67 might not be as reliable as we anticipated because cell cycle activity in adult cardiomyocytes is related to cell proliferation and polyploid state (Bersell et al., 2009). For instance, cardiomyocytes undergoing endoreduplication can also be detected with Ki67 positive without real cell division (Hesse et al., 2012), even in hypertrophy hearts (Drenckhahn et al., 2015). Thus, Ki67 can appear in both proliferative and non-proliferative cell cycles. pHH3 is a protein phosphorylated during chromatin condensation in mitosis and can evaluate mitotic activity (Hightower et al., 2012). Nevertheless, mitosis activity does not mean genuine cell division as it can result in polyploid because of cytokinesis defects (Meckert et al., 2005; Engel et al., 2006). Even Aurora B Kinase, involved in the formation of contractile rings at the region of cytoplasmic separation, is viewed as a convincing marker of cytokinesis (Crosio et al., 2002; Engel et al., 2005). Unfortunately, it is also not reliable for cell division, as various defects can still lead to cytokinesis failure and furrow regression with an Aurora B-positive midbody formation (Steigemann et al., 2009; Carlton et al., 2012). Given that no satisfying marker has yet been identified and can be used to reliably assess cell division, the published conclusions which were based on these immunohistology staining methods for evaluating different mitotic events in myocardial regeneration raise some concerns. More advanced approaches need to be developed to show authentic cardiomyocyte division for future clinical application.

### Liver polyploid and proliferation

Hepatocytes are generally quiescent with a slow renewal rate (Zajicek et al., 1985). Hepatocytes seem not fully responsive to key cytokines and growth factors in their quiescent state, such as TNF, HGF, and TGF-α, which are potent stimulators of cell cycle reentry for hepatocytes (Webber et al., 1994, 1998). Although hepatocytes are generally quiescent, they demonstrated extremely high proliferative potential upon injury (Wang et al., 2015; Chen et al., 2019). Similar to the heart arena, newly developed hepatocytes are mainly derived from pre-existing hepatocytes instead of progenitor cells (Yanger et al., 2014).

Hepatocyte ploidy state is essential in regulating liver regeneration (Wilkinson et al., 2019a). Many observations supported the assumption that the polyploid state in hepatocytes restricts their regenerative capacity (Wilkinson et al., 2019b). For example, in normal physiological conditions, hepatocytes show robust proliferative ability before weaning and this capacity gradually goes down with time; by coincidence, polyploid hepatocytes occur at weaning in rodents and increase in parallel with age (Kudryavtsev et al., 1993; Margall-Ducos et al., 2007; Celton-Morizur et al., 2009; Duncan et al., 2010). Further, liver polyploid is highly structured and proceeds more rapidly in the mid-lobule zone than in the region close to the portal and central veins, which means that the mid-lobular zone presents a higher proportion of polyploid hepatocytes compared to the portal and central veins area (Tanami et al., 2017). Two studies based on lineage tracing strategies identified a small population of hepatocytes that is capable of homeostatic liver renewal and demonstrated that these cells were mostly proximal to the central or portal veins (Font-Burgada et al., 2015; Wang et al., 2015). Notably, Wnt-responsive hepatocytes adjacent to the central vein in the liver lobule seem to be primarily diploid, responsible for proliferation and homeostatic self-renewal in the uninjured liver (Wang et al., 2015). Wilkinson et al., (2019b) found that quiescent hepatocytes were predominantly polyploid, as diploid hepatocytes can reinitiate the cell cycle and proliferate faster than polyploid hepatocytes. They determined that all ploidy populations were capable of proliferation. However, diploid hepatocytes demonstrated around a 3-fold increase in BrdU^+^ percentage compared to polyploid hepatocytes. Sigal et al., (1999) revealed that two-thirds of partial hepatectomy in rats accelerated hepatocyte senescence, attenuated proliferative capacity, and increased hepatocyte polyploidy. Earlier studies (Post and Hoffman, 1965) using tritiated thymidine incorporation into cells showed that only a few polyploid cells were labeled, displaying a prolonged S phase, which was consistent with a study showing that diploid hepatocytes have a greater capacity for DNA synthesis compared to polyploid hepatocytes (Rajvanshi et al., 1998). Although polyploid hepatocytes still maintain DNA synthesis capacity, a key concern is their declining ability to produce new cells (Gerlyng et al., 1992), which is in line with a recent study that diploid hepatocytes show higher turnover than polyploid hepatocytes (Heinke et al., 2022). Polyploid hepatocytes isolated within 5 days of partial hepatectomy are less proliferative than diploid hepatocytes (Gorla et al., 2001), suggesting that partial hepatectomy-induced polyploid hampers cell proliferation. The notion that diploid cells proliferate faster than polyploid hepatocytes is also supported by several studies which observed that diploid hepatocytes are enriched in patients and rats with various disease states (Anti et al., 1994; Rua et al., 1996; Gandillet et al., 2003).

The molecular mechanisms of why diploid hepatocytes proliferate faster than polyploid is still not well defined. In 2007, Lu et al. (2007) studied gene expression patterns between quiescent diploid and polyploid hepatocytes from mice by using a high-density genome microarray. Surprisingly, no major changes in gene expression were found, indicating that hepatocyte ploidy subsets are similar at the gene expression level. In order to test if diploid and polyploid hepatocytes had different reactions in response to hepatic mitogens, researchers treated primary wild-type hepatocytes with various growth factors related to liver regeneration. They showed diploid hepatocytes can enter and progress through the cell cycle faster, both *in vitro* and during liver regeneration *in vivo* (Rua et al., 1996).

Many mitotic hepatocytes in highly polyploid liver display extra centrosomes, indicative of polyploid hepatocyte divisions (Faggioli et al., 2011; Knouse et al., 2018; Lin et al., 2020). Researchers designed a multicolor reporter system to trace polyploid cells *in situ* and found that polyploid hepatocytes maintain extensive proliferative capacity in different liver damage models and routinely undergo reductive mitoses during the proliferation process (Matsumoto et al., 2020). Polyploid hepatocytes also showed sufficient proliferation capacity to maintain the functional integrity of livers in which more than 97% of hepatocytes were polyploid following injury (Lin et al., 2020). These polyploid hepatocytes function as efficiently as diploid hepatocytes in repairing injured organs. In Fah-deprivation mouse models, a model for selective liver replacement (Overturf et al., 1996), diploid hepatocytes were demonstrated to share equivalent regenerative capacity with polyploid hepatocytes after transplantation (Duncan et al., 2010). A similar phenomenon was also observed in the *E2f7*/*8-*deficienct liver which is composed of predominately diploid hepatocytes, and no significant difference in regenerative capacity was found between the wild-type and *E2f7*/*8-*deficienct mice (Pandit et al., 2012). Blocking centriole-mediated ploidy control causes a significantly increased hepatocyte polyploidy, severe liver damage, and impaired liver function (Sladky et al., 2022).

In order to identify whether binucleated hepatocytes can be triggered to undergo cell division or not, a live-cell imaging experiment was applied and showed that binucleated hepatocytes can stimulate cell cycle reentry and give rise to two tetraploid mononucleated daughter cells (Guidotti et al., 1909), which is similar to previous observations that binucleated hepatocytes tend to divide into two mononucleated daughter cells and thus, eventually, binucleated polyploid hepatocytes decreases and polyploid hepatocytes increases (Gerlyng et al., 1993; Miyaoka et al., 2012). Further, they applied a genetic tracing strategy and determined that merely half of the hepatocytes that undergo DNA synthesis ultimately complete the cell cycle and generate two mononuclear daughter hepatocytes (Lu et al., 2007), implying that most cell cycle activity is not authentic proliferation. Wilkinson et al., (2019b) revealed that the overall ploidy spectrum keeps unchanged after full liver regeneration in a partial hepatectomy model, which indicated that either increased ploidy is only a temporary phenomenon, ultimately adjusts itself, or probably exists a conversion of binucleated cells (2 × 2n) to mononucleated tetraploid cells (1 × 4n) after injury. Sigal et al., (1999) found a sharp decrease in the fraction of polyploid hepatocytes upon partial hepatectomy, which might attribute to the apoptotic consequence that preferentially targets polyploid hepatocytes rather than diploid cells. Conversely, in another study, diploid hepatocytes are more sensitive to apoptosis signaling compared to polyploid cells (Kreutz et al., 2017).

It has to be noticed that, in the liver field, researchers widely apply the word “regeneration” to describe the recovery of tissue weight and function, but it does not mean this process is always totally accompanied by bona fide cell proliferation. As reported previously, liver regeneration is primarily achieved through hypertrophy growth by polyploid hepatocytes without cell division after 30% partial hepatectomy. However, proliferation and hypertrophy are equally important to liver regeneration after 70% hepatectomy. Although not all polyploid hepatocytes undergo cell division, binuclear hepatocytes can still divide to increase cell numbers (Miyaoka et al., 2012). Additionally, blocking mitosis through CDK1 deletion in mouse liver does not affect hepatic regeneration after 70% partial hepatectomy, indicating that regeneration can be accomplished by hypertrophic growth of polyploid cells (Diril et al., 2012). A close parallel was found between liver mitotic index and a decrease in polyploid hepatocytes (Wheatley, 1972). As an example, the mouse liver presents a lower mitotic index level accompanied by a higher degree of hepatocytes polyploid, while the opposite occurred in the rat liver. The liver is a fascinating organ that maintains robust regenerative capacity and allows the substantial presence of polyploid and aneuploid hepatocytes. Further investigations will help disclose the polyploid hepatocyte’s biological function during liver development and regeneration.

## Conclusion

Polyploid is essential for cell growth and tissue regeneration. For instance, polyploid plays a vital role in mammal heart and liver regeneration via various cellular mechanisms and may be a promising strategy for tissue regeneration and functional recovery in clinical settings. To date, there are still several issues required to be addressed. One of the mysteries is why some polyploid cells, such as hepatocytes, can undergo successful division, whereas it is limited in others, like adult mammalian cardiomyocytes. Are there any differences in cellular levels between diploid and polyploid hepatocytes (including epigenetics, transcriptomics, and proteomics)? In addition, the advantages of multinucleated cells over mononucleated polyploid cells are still poorly understood. Perhaps an additional genome-to-nuclear surface ratio in multinucleated cells benefits cell growth or function. In the future, it will be critical to determine how the cell ploidy state is manipulated and how it affects cellular and tissue function, as a deeper understanding of polyploid will eventually benefit patients.

In short, by modulating some candidate genes or conditions, we can change polyploidy state and promote the repair and regeneration of organs such as liver and heart following injury in rodents. In this case, we believe these findings will provide a rationale for identifying therapeutic approaches to change organ ploidy state in future clinical trials, in particular in the human heart, as this might be a means to enhance heart regeneration in disease states.

## Data Availability

Data for this review manuscript was obtained from peer-reviewed publications indexed in MedLine PubMed and are all presented in this paper.

## Abbreviations

Ankrd26: ankyrin repeat domain-containing 26
Anln: anillin, actin binding protein
APC/C: anaphase-promoting complex/cyclosome
Atg7: autophagy related 7
Atp7b: ATPase copper transporting beta
CAF-1: chromatin assembly factor-1
Ccne2: cyclin E2
CDK1: cyclin-dependent kinase 1
CDKs: cyclin-dependent kinases
Csn: casein
Ect2: epithelial cell transforming 2
Ercc1: excision repair cross-complementing group 1
ESCRT-III: endosomal sorting complexes required for transport-III
Fgl2: fibrinogen like 2
Gas2l3: growth arrest specific 2 like 3
Gsk-3β: glycogen synthase kinase-3β
HGF: hepatocyte growth factor
Lkb1: liver kinase B1
Lrpprc: leucine rich pentatricopeptide repeat containing
Notch3: Notch receptor 3
P38 MAPK: P38 mitogen-activated protein kinase
PCNA: proliferating cell nuclear antigen
pHH3: phosphorylated histone H3
PIDDosom: PIDD1-RAIDD-Caspase-2 complex
Plk4: Polo like kinase 4
Rb1: retinoblastoma protein
ROS: reactive oxygen species
SAC: spindle assembly checkpoint
Skp2: S-Phase kinase-associated protein 2
TGF-α: Transforming growth factor-α
TGF-β: transforming growth factor-β
TNF: tumor necrosis factor
Trf2: telomeric repeat binding factor 2
